# Genome-Wide Association Study Identifies Quantitative Trait Loci and Candidate Genes Involved in Deep-Sowing Tolerance in Maize (*Zea mays* L.)

**DOI:** 10.3390/plants13111533

**Published:** 2024-06-01

**Authors:** Jin Yang, Zhou Liu, Yanbo Liu, Xiujun Fan, Lei Gao, Yangping Li, Yufeng Hu, Kun Hu, Yubi Huang

**Affiliations:** 1 State Key Laboratory of Crop Gene Resource Exploration and Utilization in Southwest China, Sichuan Agricultural University, Chengdu 611130, China; cilantro_j@hotmail.com (J.Y.); zhoul163@163.com (Z.L.); bbwalk3r@163.com (Y.L.); 2020301100@stu.sicau.edu.cn (X.F.); gaolei@sicau.edu.cn (L.G.); ypli@sicau.edu.cn (Y.L.); huyufeng@sicau.edu.cn (Y.H.); 2 Sinograin Chengdu Storage Research Institute Co., Ltd., Chengdu 610091, China

**Keywords:** maize, deep sowing, genome-wide association study, quantitative trait locus, *ZmGCP2*, plumule length, shoot length

## Abstract

Deep sowing is an efficient strategy for maize to ensure the seedling emergence rate under adverse conditions such as drought or low temperatures. However, the genetic basis of deep-sowing tolerance-related traits in maize remains largely unknown. In this study, we performed a genome-wide association study on traits related to deep-sowing tolerance, including mesocotyl length (ML), coleoptile length (CL), plumule length (PL), shoot length (SL), and primary root length (PRL), using 255 maize inbred lines grown in three different environments. We identified 23, 6, 4, and 4 quantitative trait loci (QTLs) associated with ML, CL, PL, and SL, respectively. By analyzing candidate genes within these QTLs, we found a γ-tubulin-containing complex protein, *ZmGCP2*, which was significantly associated with ML, PL, and SL. Loss of function of *ZmGCP2* resulted in decreased PL, possibly by affecting the cell elongation, thus affecting SL. Additionally, we identified superior haplotypes and allelic variations of *ZmGCP2* with a longer PL and SL, which may be useful for breeding varieties with deep-sowing tolerance to improve maize cultivation.

## 1. Introduction

Maize (*Zea mays* L.) is one of the most productive and high-value crops worldwide and plays a pivotal role in global food security [1]. Achieving uniform and vigorous seedling emergence is essential for increasing maize productivity [2]. Maize emergence is influenced by a multitude of factors including genetic variation in seeds, environmental factors, planting depth, and sowing techniques. Adverse climatic conditions, such as drought and low temperatures, are critical factors limiting maize emergence rates and seedling quality [3,4,5]. However, increasing sowing depth can effectively mitigate the negative effects of environmental stresses such as drought and low temperatures on maize seedling growth [6,7,8,9]. Furthermore, deep sowing can promote the development of deep root systems in maize, which are vital for the later stages of water absorption and utilization in plants; as the root system extends into deeper soil layers, maize plants use deep-soil water resources more effectively and exhibit greater resistance to lodging [10]. Since the 1990s, the Indian maize variety “P1213733 (Komona)” has been widely cultivated in the dryland areas of southwestern United States and western Mexico, because of its ability to be sown at a depth of 30 cm and maintain good emergence rates [11]. In China, through the genetic enhancement of Indian blue corn, breeders have developed maize varieties such as “42107” and “Kang 42”, which not only adapt well to deep-sowing conditions but also exhibit improved drought tolerance and yield [10].

Owing to genetic differences among different varieties of seeds, increasing sowing depth may result in some seeds failing to emerge properly [12]. Therefore, to improve maize emergence and yield through deep sowing, maize seeds should have an elevated level of deep-sowing tolerance, which entails maintaining a high germination rate and good seedling vigor under deep-sowing conditions. Many studies have evaluated the deep-sowing tolerance of maize by measuring traits such as germination rate, mesocotyl length (ML), coleoptile length (CL), plumule length (PL), shoot length (SL), and primary root length (PRL) [13,14,15,16]. Among these traits, the emergence rate is the most straightforward metric for assessing the deep-sowing tolerance of maize, with higher rates corresponding to increased tolerance. In deep-sowing conditions, the emergence rate is primarily and directly correlated with ML, followed by CL and SL [17]. Longer plumules and shoots also indicate the greater vitality of seeds and seedlings [18].

Many deep-sowing tolerance-related quantitative trait loci (QTLs) have been mapped using different populations of rice (*Oryza sativa* L.) [19,20,21,22], and the functions of several genes related to the deep-sowing tolerance of rice have been reported [23,24,25]. In contrast, only a limited number of deep-sowing tolerance QTLs have been identified in maize [11,17,26]. These maize QTLs predominantly stem from biparental population studies constrained by a limited number of molecular markers. This limitation restricts the precision of QTL localization, often confining QTLs to broader genomic regions, making further mining of candidate genes difficult. Consequently, some studies have combined RNA sequencing and meta-analysis to better dissect the genetics of deep-sowing tolerance in maize. Zhao et al. [16] identified 13 major QTLs and 17 meta-QTLs associated with deep-sowing tolerance, along with 50 differentially expressed candidate genes within these QTLs. Wang et al. [27] used transcriptome analysis to elucidate the molecular mechanisms of maize seed germination under deep-sowing conditions, identifying six candidate genes. In the study of quantitative traits in plants, genome-wide association studies (GWAS) use dense genetic markers to achieve a more precise association with genetic areas relevant to target traits, in contrast to traditional mapping methods [28,29,30]. This method is particularly useful for detecting loci with minor effects, thereby substantially advancing the elucidation of complex trait genetics and the discovery of candidate genes [31,32,33,34,35]. Yang et al. [9] identified 273 single nucleotide polymorphisms (SNPs) significantly associated with deep-sowing tolerance in maize through GWAS and RNA sequencing, selecting one candidate gene related to organ length, auxin, or light response. In summary, these studies have enhanced the comprehension of the genetics underlying maize deep-sowing tolerance. However, the number of high-confidence candidate genes identified in current research on maize deep-sowing tolerance remains limited, and the functions of these genes require further investigation.

In this study, the association population was re-sequenced, and high-density and high-quality SNP markers were obtained. Based on this premise, we performed a genome-wide association analysis of deep-sowing tolerance-related traits in maize, including ML, CL, PL, SL, and PRL. We identified several QTLs associated with deep-sowing tolerance in maize co-localized with the candidate gene *ZmGCP2*, which was significantly associated with ML, PL, and SL. We then verified the function of *ZmGCP2* using an ethyl methanesulfonate (EMS) mutant. We found that *ZmGCP2* likely alters PL through its effect on cell elongation, thereby affecting SL. Furthermore, we identified superior haplotypes and allelic variations in *ZmGCP2* that may be useful for breeding varieties with deep-sowing tolerance to improve maize cultivation.

## 2. Results

### 2.1. Evaluation of Deep-Sowing Tolerance-Related Traits

In the present study, deep-sowing tolerance-related traits were evaluated in an association population grown in three different environments: Chongzhou in 2020 (20CZ), Yunnan in 2020 (20YN), and Chongzhou in 2021 (21CZ) (Table 1). All traits were measured in maize seedlings that had been grown in the dark for 8 d. Abundant phenotypic variations were observed in ML, CL, PL, SL, and PRL, and the results indicated this population was suitable for analyzing the genetic basis of deep-sowing tolerance-related traits (Table 1). The absolute values of skewness and kurtosis for each trait were less than one, indicating that they were normally distributed, which is a typical characteristic of quantitative traits (Table 1). The broad-sense heritability for each trait ranged from 0.82 to 0.91, indicating that deep-sowing tolerance-related traits in maize were mainly influenced by genetic factors (Table S1). Additionally, Pearson correlation analysis of both deep-sowing tolerance-related traits and grain traits (kernel length, kernel width, kernel thickness, hundred-grain weight, area of endosperm, and area of embryo) revealed that ML and PL were significantly positively correlated with SL, whereas the other traits were not significantly correlated (Figure S1). This finding indicates that traits related to deep-sowing tolerance were not affected by grain traits.

### 2.2. Linkage Disequilibrium and Structure Analysis

**Figure 1:**
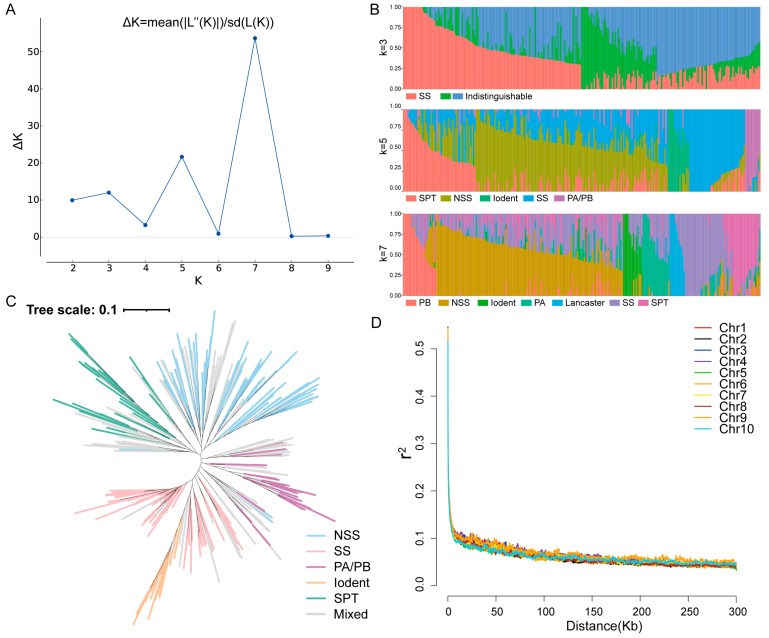
Linkage disequilibrium (LD) and structure analysis of the association population. (**A**) Values of ΔK plotted against K = 1–10 based on the structure analysis of the accessions population. (**B**) Population structure with K  =  3, 5, and 7. (**C**) Phylogenetic tree of the entire panel for K  =  5. (**D**) LD decay across the 10 maize chromosomes. Chr1–Chr10 represent chromosomes 1–10.

The population structure of the association panel was subsequently identified. According to the ∆K calculation results, coupled with breeding experience and pedigree information, the association population could be divided into three, five, or seven groups (Figure 1A). When K = 3, the SS group was clearly separated; however, the other two groups were more difficult to distinguish. For instance, when K = 5, the entire population could be divided into SS, NSS, PA/PB, Iodent, and SPT groups. The PA and PB groups could be separated further, and the Lancaster group could also be divided when K = 7 (Figure 1B). Inbred lines with lower discernibility were allocated to the mixed group. Based on the population division results, we constructed a phylogenetic tree for K = 5 (Figure 1C). The division of different branches within the entire phylogenetic tree was similar to the division of the population structure, and the mixed group was predominantly located in transitional areas between the other groups. Furthermore, linkage disequilibrium (LD) analysis of SNP markers across 10 chromosomes indicated that at a cutoff of r^2^ = 0.1, the decay distance for LD was approximately 10 kb (Figure 1D). Therefore, the LD threshold used in this research was 10 kb.

### 2.3. GWAS of Deep-Sowing Tolerance-Related Traits in Maize

**Figure 2:**
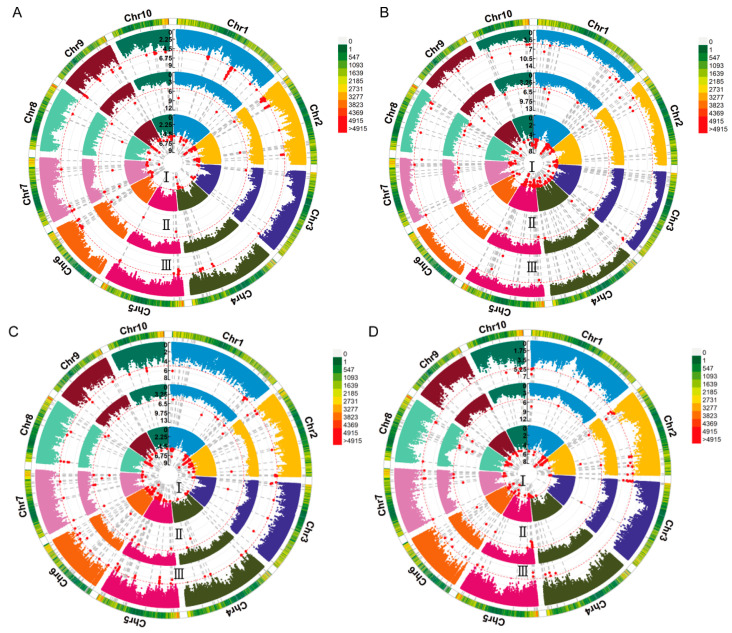
Circular Manhattan plot of the genome-wide association study (GWAS) for deep-sowing tolerance-related traits. (**A**–**D**) Manhattan plots of ML, CL, PL, and SL, respectively. ML, mesocotyl length; CL, coleoptile length; PL, plumule length; SL, shoot length. I–III represent different environments: (I) 2020 Chongzhou, Sichuan; (II) 2020 Xishuangbanna, Yunnan; (III) 2021 Chongzhou, Sichuan. Chr1–Chr10 represent chromosomes 1–10. The red line represents the significance threshold of *p* = 1 × 10^−5^. The red dots indicate single nucleotide polymorphisms (SNPs) significantly associated with deep-sowing tolerance-related traits.

**Figure 3:**
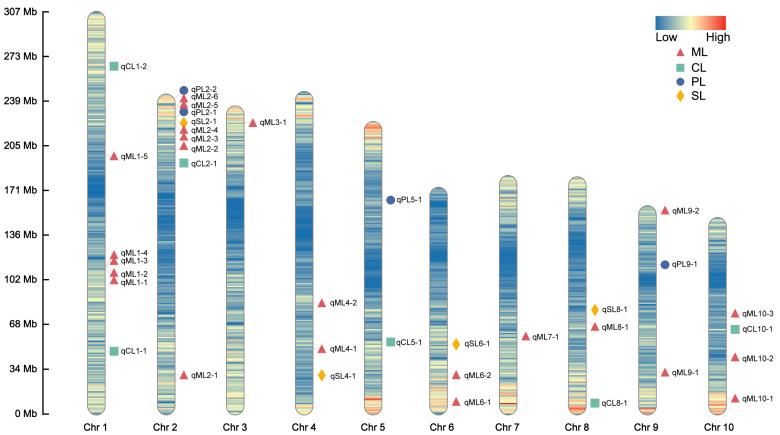
Chromosomal distributions of the quantitative trait loci (QTLs) for deep-sowing tolerance-related phenotypes in the association population. Chr1–10 represent chromosomes 1–10. The color of the chromosome represents the density of SNPs. The QTL names are indicated according to the following rules: the chromosome number is indicated by the first number following the phenotype name, and the number following the dash is used to identify different QTLs found on the same chromosome for the same trait.

A GWAS of deep-sowing tolerance-related traits was conducted in an association population grown across three environments. The association analysis was performed using the FarmCPU model, with a significance threshold of *p* = 1 × 10^−5^. We identified 38, 6, 4, and 5 SNPs that were significantly associated with ML, CL, PL, and SL, respectively, in at least two environments, each of which we referred to as co-located SNPs (Figure 2 and Figure S2). No significant SNP for PRL was consistently detected in multiple environments (Figure S3). The LD region (10 kb) around these co-located SNPs was defined as a QTL, and the QTLs that overlapped and were related to the same trait were combined into a single QTL. According to this, 23, 6, 4, and 4 QTLs were associated with ML, CL, PL, and SL, respectively. These QTLs were distributed over 10 chromosomes, most of which were located on chromosome 2 (Figure 3). The phenotypic variance explained by these QTLs was mostly less than 10%, indicating that deep-sowing tolerance-related traits were mainly controlled by multiple minor-effect loci (Table S2). Of particular interest were the qML2-3, qPL2-2, and qSL2-1 loci on chromosome 2, which exhibited overlapping regions.

### 2.4. Candidate Genes Co-Localized by ML, PL, and SL

Within the LD = 10 kb region of significant SNPs, 43, 13, 16, and 10 candidate genes were identified for ML, CL, PL, and SL, respectively, across two environments (Table S3). Additionally, we found that *Zm00001d002644* and *zma-MIR171h* were associated with ML, PL, and SL in different environments. Pearson correlation analysis of deep-sowing tolerance-related traits revealed a strong positive correlation between ML and PL with SL, suggesting interconnected growth processes. *Zm00001d002644* encodes the Spc97/Spc98 family of spindle pole body (SBP) components, which are part of the γ-tubulin complex. Microtubules are the main components of the cytoskeleton and are formed by the polymerization of tubulin dimers, which play important roles in maintaining cell morphology, activity, and division [36]. *zma-MIR171h* was reported to potentially regulate the response to low phosphorus and cadmium stresses in maize and may be associated with the interaction between maize and the pathogen *Fusarium verticillioides* [37,38,39]. Based on the reported expression data [40,41], we analyzed the expression level of *Zm00001d002644* and *zma-MIR171h* (Figure S4). The results showed that *Zm00001d002644* was highly expressed in the coleoptile 6 days after sowing, which included the mesocotyl, coleoptile, and plumule in the early growth stage. In addition, it was also highly expressed in the fast-growth tissues, such as the shoot apical meristem, shoot tip, and primary root. *zma-MIR171h* was highly expressed in the leaf and seedling but showed lower expression in other tissues. Therefore, we focused on *Zm00001d002644* and subsequently named it gamma-tubulin complex protein 2 (*ZmGCP2*).

### 2.5. Functional Verification of Candidate Gene ZmGCP2

**Figure 4:**
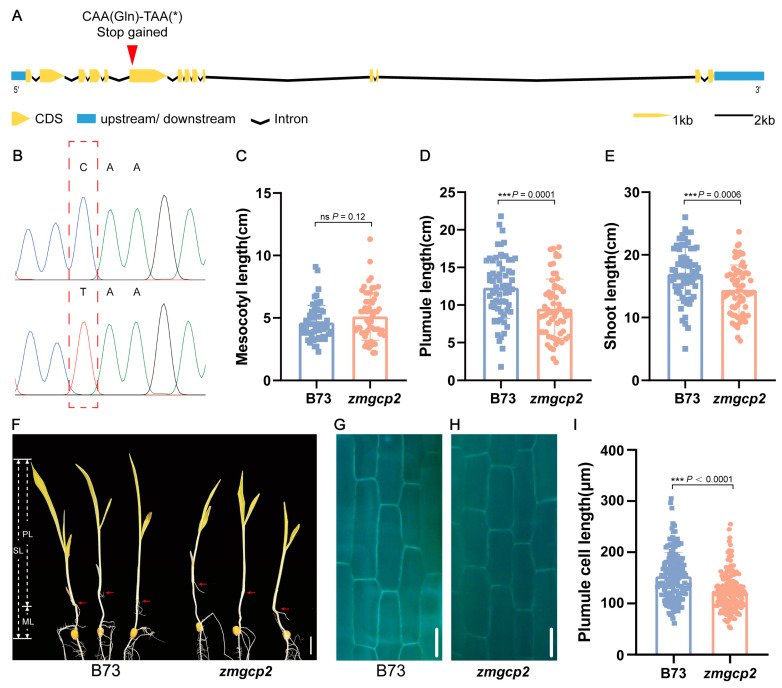
Mutation and phenotypes of *zmgcp2* compared to wild-type B73. (**A**) Schematic charts of *ZmGCP2* gene structure; asterisk stands for stop codon. The red arrowhead indicates the mutation position in *zmgcp2*. (**B**) Sequencing results of B73 and *zmgcp2* at the mutation position. (**C**–**E**) Comparison of ML, PL, and SL between B73 and *zmgcp2*. (**F**) Seedling phenotypes of B73 and *zmgcp2*; scale bars = 2 cm. (**G**–**H**) Plumule cells of B73 and *zmgcp2* under blue excitation light; scale bars = 50 μm. (**I**) Comparison of plumule cell length between B73 and *zmgcp2*; n ≥ 90 cells from 3 seedlings. Student’s *t*-test; ***, *p* < 0.001; ns, not significant.

To functionally characterize *ZmGCP2*, we obtained a B73 EMS mutant (*zmgcp2*) from the EMS mutant library, which contained a termination mutation in the sixth exon (Figure 4A,B). Comparative phenotypic assessments of *zmgcp2* and wild-type B73 revealed no difference in ML, whereas PL and SL were significantly shorter in *zmgcp2* than in B73 (Figure 4C–F). Additionally, we observed the plumule cells of the mutant *zmgcp2* and B73 and found that the plumule cell length of *zmgcp2* was significantly shorter than that of B73 (Figure 4G–I). These findings suggest that *ZmGCP2* is a candidate gene that alters PL by affecting cell elongation, thereby affecting SL.

### 2.6. Natural Variation in ZmGCP2

**Figure 5:**
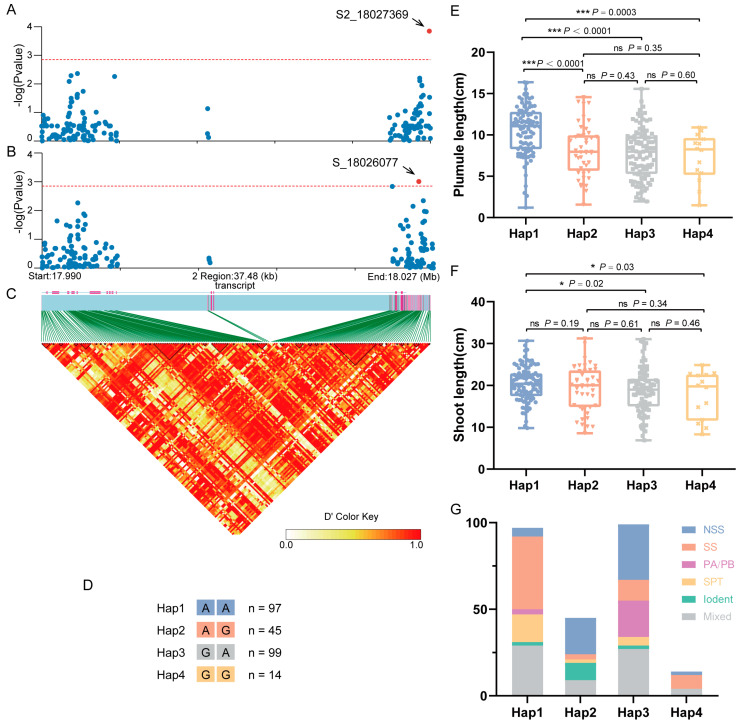
Association analysis of the candidate gene *ZmGCP2*. (**A**) Candidate gene association analysis of *ZmGCP2* with PL-best linear unbiased estimate (BLUE). (**B**) Candidate gene association analysis of *ZmGCP2* with SL-BLUE. (**C**) Pairwise LD between the markers. (**D**) Details of four haplotypes (Hap1, Hap2, Hap3, and Hap4); n represents the inbred line number of each haplotype. (**E**,**F**) Comparison of PL and SL between different haplotypes. Student’s *t*-test; *, *p* < 0.05; ***, *p* < 0.001; ns, not significant. (**G**) Distribution of inbred lines from different groups in the four haplotypes.

An association analysis was performed on SNPs within the coding sequence (CDS) and 2000 bp upstream/downstream regions of *ZmGCP2* based on the PL-best linear unbiased estimate (BLUE) and SL-BLUE values. PL and SL were significantly associated with S2_18027369 and S_18026077, respectively, with S2_18027369 located in the downstream regions of the CDS and S_18026077 located on the exon (Figure 5A–C). Based on the allelic types at these two loci, the association population was divided into four haplotypes (Hap1: AA; Hap2: AG; Hap3: GA; and Hap4: GG) (Figure 5D). Compared to the other three haplotypes, Hap1 exhibited significantly higher PL and SL, with SL being significantly higher than in Hap3 and Hap4, but not significantly higher than in Hap2 (Figure 5E,F). Thus, we deduced that Hap1 was the superior haplotype and that allele A at S_18026077 (S_18026077^-A^) was the superior allelic variation for controlling PL and SL. Moreover, we analyzed the distribution of different groups of inbred lines among the four haplotypes. The results showed a high prevalence of the SS group within Hap1, followed by the Tang Si Ping Tou (SPT) hybrid advantage group in China. The NSS group and other groups were very limited (Figure 5G). These findings led us to hypothesize that this gene has undergone selection during maize improvement and produces a favorable Hap1 allele that may be useful and suitable for modern breeding programs for maize improvement.

## 3. Discussion

### 3.1. GWAS Is Effective for Dissecting the Genetic Basis of Maize Deep-Sowing Tolerance

Uniform and vigorous seedling emergence is an important precondition for improving maize yield [2]. Adverse climatic conditions are important limiting factors for the emergence rate and seedling quality of maize [3,4,5]. Deep sowing can effectively mitigate the negative effects of environmental stresses, such as drought and low temperatures, on the growth of maize seedlings [6,7,8,9]. Furthermore, deep sowing can promote the development of deep root systems and drought and lodging resistance in maize [10]. To increase the production and emergence rate of maize via deep sowing, maize seeds should have an elevated level of deep-sowing tolerance. Genetic analysis and candidate gene mining for deep-sowing tolerance-correlated maize traits can benefit the cultivation of deep-sowing and drought-tolerant maize varieties. Currently, the number of QTLs for deep-sowing tolerance that have been found in maize is limited [11,17,26]. These QTLs are mostly obtained from biparental populations with broad genetic distances, making it difficult to extract candidate genes for use in breeding programs.

GWAS are based on the LD within populations that exhibit wide natural genetic variations. GWAS use statistical methodologies to analyze the relationships between genotypic variations and targeted traits. They have been widely applied to investigate the genetic basis of complex traits and identify SNPs [28,29,30,31,32,33,34,35]. In this study, multiple traits related to deep-sowing tolerance were mapped and genetic variation was analyzed simultaneously using a GWAS. The variability coefficients of deep-sowing tolerance-related traits of the association population ranged from 24% to 40%, which shows that this population had diverse genetic variation and was suitable for analyzing the genetic basis of deep-sowing tolerance-related traits. High-density molecular markers are crucial for identifying superior genetic variants; in this study, 2,432,795 markers were obtained by 10× resequencing of the association population. Based on these results, a genetic structure analysis was performed, and genetic location relevance and candidate gene identification were ensured.

### 3.2. Comparison of Co-Localized QTLs with Previously Reported QTLs

In the present study, ML, CL, PL, and SL were associated with 38, 6, 4, and 5 significant SNPs, respectively, which were each repeatedly detected in at least two environments. Defining regions by co-localized SNPs ± LD (10 kb), we identified 23, 6, 4, and 4 corresponding QTLs. Zhang et al. [17] identified several deep-sowing tolerance-related QTLs at sowing depths of 10 and 20 cm in the F_2_ population. The qML4-2, qML6-1, qML6-2, and qML10-3 loci identified in our study overlapped with the qMES20-4, qMES10-6-2, and qMES10-10 loci identified by Zhang et al. [17], and these QTLs were all related to ML. Additionally, qML3-1 for ML overlapped with qSDL20-3 for SL in the same study. Simultaneously, the qML4-1 locus in our study overlapped with qDSSL4-2, which is associated with SL under deep-sowing conditions in the IBM Syn10 DH population, as reported by Liu et al. [26]. Furthermore, the qSL4-1 and qSL6-1 loci, which are related to SL, overlapped with qMES20-4 and qMES10-6-2, which were found to control ML by Zhang et al. [17]. According to the correlation analysis between each trait, ML and SL were significantly and positively correlated; therefore, detecting the same QTL interval between ML and SL was reasonable. Finally, the qCL1-2 locus for CL on Chr1 overlapped with the germination rate QTL qRAT20-1 found at the 20 cm sowing depth and was close to qRAT10-1 at the 10 cm depth, both detected by Zhang et al. [17]. It was also near qDSGR1-1, which was found to be related to germination rate in a study by Liu et al. [26]. In conclusion, the QTLs identified in this study are valid and reliable. Many of these QTLs are novel, providing new information for the genetic analysis of deep-sowing tolerance in maize. The use of high-density SNP markers in this study led to the identification of QTL intervals that were significantly smaller than those found in similar research efforts, which improves the possibility of further mining candidate genes. Moreover, because of genetic variations in different populations, the QTL intervals associated with the same traits will differ, and different traits with correlations are likely to be associated with the same QTL interval.

### 3.3. Functional Analysis of Candidate Gene ZmGCP2

Under deep-sowing conditions, seeds can maintain a high germination rate, and strong seedling vigor is an important manifestation of their tolerance to deep sowing. The emergence rate of maize under deep-sowing conditions is significantly correlated with ML [13,14,15,16], followed by PL and SL [17]. In fact, longer plumules and shoots also indicate greater vitality of seeds and seedlings [18]. In this study, ML and PL were significantly and positively correlated with SL, and we co-localized a candidate gene, *ZmGCP2*, which was significantly associated with ML, PL, and SL. *ZmGCP2* encodes the Spc97/Spc98 family of SBP components, which are part of the γ-tubulin complex. Microtubules are the main components of the cytoskeleton that exist in all eukaryotic cells and are highly conserved. Tubulin plays important roles in the maintenance of cell morphology, intracellular material transport, cell activity, signal transduction, and cell division during plant growth and development [36]. During cell division, tubulin is crucial in determining the location of cell division and promoting the separation of the nucleus. Additionally, tubulin can affect cellulose deposition by changing its orientation, thus inhibiting cell elongation [42,43,44,45]. In rice, the arrangement of cortical microtubules in the *OsTubA1* mutant *wg4-D* is altered, leading to smaller and shorter cells in the outer lemma, resulting in increased grain width and decreased grain length and 1000-grains weight [46]. Overexpression of the microtubule-associated protein *ZmRPH1* from the QWRF family in maize results in shorter and broader mesocotyls, reduced plant height, and decreased root length [47].

In this study, we conducted a phenotypic assessment of a *ZmGCP2* mutant with the premature termination of transcription. The findings showed no significant difference in ML between the homozygous mutant and wild type; however, both PL and SL were significantly reduced. The QTL related to *ZmGCP2* in the GWAS accounted for approximately 11.81% of the variation in PL and a slightly lower contribution to ML (approximately 8.52%). Therefore, a lower effect may have led to a lack of significant changes in the mesocotyl phenotype of the mutant. Additionally, the plumule cell length was significantly shorter than in wild-type B73 cells. Therefore, we consider *ZmGCP2* a candidate gene for PL and SL, likely altering PL through its effect on cell elongation, thereby affecting SL.

Numerous studies have successfully identified superior allelic variants of relevant genes through candidate gene association analyses, which have been integrated into various genetic backgrounds, demonstrating their potential utility in breeding [25,48,49]. In this study, we conducted a candidate gene association analysis of the key gene *ZmGCP2* identified in our GWAS. The results showed that Hap1 was the superior haplotype that influenced PL and SL, and S_18026077^-A^ was the superior allelic variation for controlling PL and SL. Moreover, we observed a high prevalence of the SS group within Hap1, followed by the SPT hybrid advantage group in China, with that of the NSS group and other groups being very limited. These findings led us to hypothesize that this gene has undergone selection during maize improvement and produces a favorable Hap1 allele that may be useful and suitable for modern breeding programs for maize improvement.

## 4. Materials and Methods

### 4.1. Plant Materials and Growth Conditions

The plant materials used in this study included a maize-association population and EMS mutants. The association population was composed of 255 maize inbred lines, which were used to assess deep-sowing tolerance-related traits in our GWAS. These lines were kindly provided by Professor Zuxin Zhang of Huazhong Agricultural University, Wuhan, China. The pedigree and source information of inbred lines in the association population was listed in Table S4. The association population was planted in three different environments: 20CZ (2020 Chongzhou, Sichuan), 20YN (2020 Xishuangbanna, Yunnan), and 21CZ (2021 Chongzhou, Sichuan). Each line was harvested at full maturity and subsequently used for measuring deep-sowing tolerance-related traits after sufficient drying. The EMS mutants were obtained from the maizeEMSDB mutant library (http://maizeems.qlnu.edu.cn/, accessed on 18 March 2022) [50] and cultivated on a farm at Sichuan Agricultural University, Chongzhou, Sichuan.

### 4.2. Phenotyping and Statistical Analysis

In the present study, 18 seeds of similar size and disease-free status were selected from each of the 255 maize inbred lines. The seeds were soaked in sterile water for 24 h and then wrapped into rolls in germination paper (Anchor Paper, St. Paul, MN, USA) following the methodology described by Abdel et al. [51]. Briefly, six seeds were placed 6 cm away from the top edge of a double layer of germination papers that had been pre-moistened with sterile water. Seeds were placed 3.5 cm apart and 3.5 cm from the left and right edges, covered with another germination paper, and then wrapped into rolls about 5 cm thick and secured with a rubber band. The rolls were placed vertically in black plastic containers filled with 500 mL of sterile water, and each container was covered with a black plastic bag to ensure darkness and minimize moisture loss. The containers were placed in an incubator at 26 °C with 65% relative humidity in darkness for 8 d. Then, the ML, CL, PL, SL, and PRL of each line were measured three times.

Descriptive statistical analyses of each trait, including range, mean, standard deviation (SD), coefficient of variation (CV), skewness, and kurtosis, were performed using IBM SPSS Statistics 26 software. Broad-sense heritability was calculated using the lme4 package in R (version 4.2.2) with the following Equation (1):

(1) $$H^{2}=\frac{Vg}{Vg+\frac{Vge}{L}+\frac{Vgy}{Y}+\frac{Ve}{YRL}}$$

where *Vg* is the variance of genotype, *Ve* is the variance of residual, *Vge* is the interaction of genotype and environment variance, *Vgy* is the interaction of genotype and year variance, *L* is the number of environments, *Y* is the number of years, *YRL* is number of years × number of repeats × number of environments, and *H*^2^ is the broad-sense heritability of a trait. Correlations between traits were analyzed and visualized using the GGally extension (version 2.2.0) of ggplot2 (version 3.4.4). Other statistical analyses and visualizations were performed using the GraphPad Prism 8 software.

### 4.3. SNP Genotyping

DNA extraction and library construction for sequencing were performed by Chengdu Tiancheng Future Technology Co., Ltd., Chengdu, China. The sequencing results were aligned to the B73 RefGen_v4 (https://download.maizegdb.org/Zm-B73-REFERENCE-GRAMENE-4.0/Zm-B73-REFERENCE-GRAMENE-4.0.fa.gz, accessed on 16 March 2021) reference genome using BWA software (version 0.7.17-r1188) for indexing. Each sequencing file was mapped, and the format was converted using SAMtools (version 1.7). SNP calling was performed using BCFtools (version 1.9) and multiple VCF files were merged. Finally, VCFtools (version 0.1.16) was used to filter out SNPs with a minor allele frequency < 0.05, missing rate > 20%, or heterozygosity > 30%.

### 4.4. LD and Structure Analysis

FastStructure was used for structural analysis, with K values ranging from 1 to 10 [52]. The ggplot2 (version 3.4.4) package in R (version 4.2.2) was used to generate stacked plots of the associated population structure. The most probable number of groups was determined by calculating the ΔK value with STRUCTURE 2.3.4 software [53]. Genetic distances between lines were calculated using the SNPhylo package (version 20180901) [54] and the resulting tree files were imported into the ITOL website (https://itol.embl.de/, accessed on 9 March 2024) for visualization. According to the ∆K calculation results, and coupled with breeding experience and pedigree information, the associated population was divided into different groups [55,56,57]. The LD analysis of SNP markers across the chromosomes was performed using PopLDdecay (version 3.42) [58]. The LD decay distance was defined as the interval containing an r^2^ value of less than 0.1 (LD = 10 kb).

### 4.5. GWAS

The GWAS was conducted using the FarmCPU model within the rMVP package (version 1.0.6) in R (version 4.2.2) [59], with a significance threshold of *p* = 1 × 10^−5^. Significant SNPs identified in at least two environments were referred to as co-located SNPs. The LD region (10 kb) around the co-located SNPs was defined as a QTL, and the QTLs that overlapped and were related to the same trait were combined into a single QTL. All gene models within the LD = 10kb regions of significant SNPs were identified as initial candidate genes from the GWAS. Candidate genes were functionally annotated by referring to B73 RefGen V4 in MaizeGDB (https://www.maizegdb.org/, accessed on 2 January 2024).

### 4.6. Gene Expression Patterns Analysis

We used reported expression data to analyze the expression patterns of candidate genes *Zm00001d002644* and *zma-MIR171h*. The expression data for *Zm00001d002644* were obtained from Schaefer et al. [40], and the expression data for *zma-MIR171h* were downloaded from the website https://www.pmiren.com/ (accessed on 15 May 2024) [41].

### 4.7. Candidate Gene Association Study

The BLUE value of the traits was used to conduct a candidate gene association analysis of SNPs within the CDS and 2000 bp upstream/downstream regions of a candidate gene. In the candidate gene association analysis, the General Linear Model available in TASSEL 5.0 [60] was used. The significance threshold was set to *p* = 1/N, where N represents the number of markers within a specified range. The haplotypes were divided according to the allele type of the significant SNPs. Subsequently, a Student’s *t*-test was conducted to evaluate the phenotypic variations among these haplotypes, enabling the identification of the most advantageous haplotype. Statistical significance was set at *p* < 0.05.

### 4.8. Cell Observation

The plumule cells were stained with calcofluor white (SL7204; Coolaber Technology Co., Ltd., Beijing, China) and observed under blue light excitation; calcofluor white is a nonspecific fluorescent stain that binds to cellulose and chitin in the cell walls of fungi and other organisms. Briefly, a 1 cm section was taken from the middle of uniformly growing plumules (the first true leaf) and placed on a microscope slide. A drop of the calcofluor white stain was added, and a drop of 10% potassium hydroxide was applied before the slide was covered with a coverslip. After staining for 1 min, cells were directly observed and photographed under a fluorescence microscope (CKX53; Olympus Corporation, Tokyo, Japan). The length of the cells was measured using ImageJ software (version 1.53a). Each sample involved measurements of no fewer than 30 cells, with at least three biological replicates used for statistical analysis.

## 5. Conclusions

In this study, 255 maize inbred lines were used to perform a GWAS on traits related to deep-sowing tolerance. We identified 23, 6, 4, and 4 QTLs associated with ML, CL, PL, and SL, respectively, and co-localized a candidate gene, *ZmGCP2*, which was significantly associated with ML, PL, and SL. Subsequent functional validation of *ZmGCP2* in maize EMS mutants suggested that *ZmGCP2* likely alters PL through its effect on cell elongation, thereby affecting SL. Additionally, we identified superior haplotypes and allelic variations of *ZmGCP2* with a longer PL and SL, which may be useful for breeding varieties with deep-sowing tolerance to improve maize cultivation.

## Figures and Tables

**Table 1:** Descriptive statistics for the deep-sowing tolerance-related traits of association population grown in three different environments.

Env.	Traits	Range (cm)	Mean (cm)	SD	CV%	Skewness	Kurtosis
20ZC	ML	3.25–21.74	10.47	3.40	31.67%	0.53	0.16
	CL	1.25–7.78	4.59	1.09	24.26%	−0.13	0.33
	PL	1.10–16.35	9.16	3.49	38.14%	−0.07	−0.63
	SL	5.13–33.37	19.44	5.14	26.44%	−0.17	0.04
	PRL	4.85–36.85	23.95	6.53	27.27%	−0.48	−0.28
20YN	ML	3.00–24.25	10.74	3.59	33.43%	0.66	0.74
	CL	1.40–8.02	4.51	1.08	23.88%	0.15	0.67
	PL	1.4–17.38	8.84	3.43	38.80%	−0.04	−0.66
	SL	6.03–33.83	19.48	5.45	27.96%	−0.10	−0.31
	PRL	3.20–40.10	24.48	7.38	30.14%	−0.48	0.10
21CZ	ML	2.70–22.03	10.79	3.72	34.47%	0.40	−0.08
	CL	1.52–7.57	4.44	1.03	23.19%	−0.17	0.24
	PL	1.20–17.75	8.97	3.66	40.73%	−0.10	−0.55
	SL	4.70–31.68	19.30	5.35	27.74%	−0.30	−0.22
	PRL	2.00–40.00	25.38	7.38	29.07%	−0.71	0.05

20CZ, 2020 Chongzhou, Sichuan; 20YN, 2020 Xishuangbanna, Yunnan; 21CZ, 2021 Chongzhou, Sichuan. ML, mesocotyl length; CL, coleoptile length; PL, plumule length; SL, shoot length; PRL, primary root length; Env., environments; SD, standard deviation; CV, coefficient of variation. Skewness measures the asymmetry of the distribution around its mean. Kurtosis indicates the tailedness of the distribution, showing how peaked or flat it is compared to a normal distribution.

## Data Availability

The data presented in this study are available on request from the corresponding author. The data are not publicly available due to restrictions on protected maize lines.

## Supplementary Materials

The following supporting information can be downloaded at: https://www.mdpi.com/article/10.3390/plants13111533/s1, Figure S1: Pearson correlation analysis of both deep-sowing tolerance-related traits and grain traits; Figure S2: Q-Q plots of the GWAS for deep-sowing tolerance-related traits; Figure S3: Circular Manhattan and Q-Q plots of the GWAS for PRL; Figure S4: Expression pattern analysis of the candidate genes; Table S1: Variance analysis for deep-sowing tolerance-related traits in association populations under three environments; Table S2: QTLs identified in association population by a GWAS; Table S3: List of candidate genes; Table S4: Pedigree and source information of inbred lines in association population.

## Abbreviations

Iodent	Iowa Experiment Station Reid Yellow Dent
NSS	Non-Stiff Stalk
PA	Group A germplasm derived from modern U.S. hybrids
PB	Group B germplasm derived from modern U.S. hybrids
SPT	Si Ping Tou
SS	Stiff Stalk
