# Supplementary material for: Genome-Wide Association Study Identifies Quantitative Trait Loci and Candidate Genes Involved in Deep-Sowing Tolerance in Maize (Zea mays L.)
Source: Plants (Basel). 2024 Jun 1;13(11):1533. doi: 10.3390/plants13111533 (PMC11175157; doi:10.3390/plants13111533)
Supplement: Supplementary file 1 [file plants-13-01533-s001.zip › plants-2998408-supplementary.pdf]

Supplementary Materials

FIGURES:

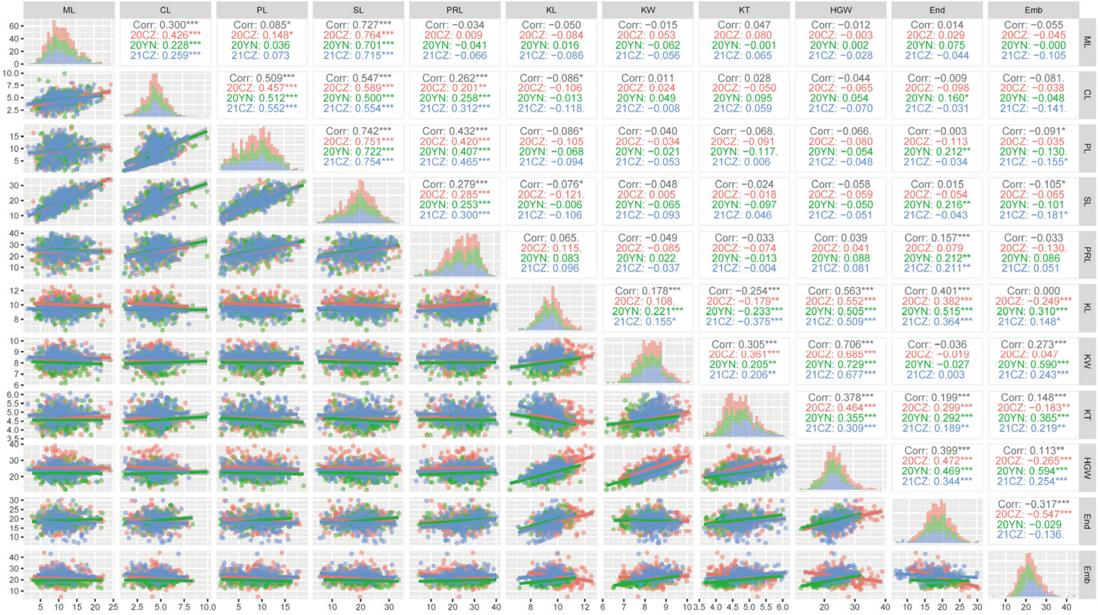

Figure S1. Pearson correlation analysis of both deep-sowing tolerance-related traits and grain traits. 20CZ, 2020 Chongzhou, Sichuan; 20YN, 2020 Xishuangbanna, Yunnan; 21CZ, 2021 Chongzhou, Sichuan; CL, coleoptile length; Emb, area of embryo; End, area of endosperm; HGW, hundred-grain weight; KL, kernel length; ML, mesocotyl length; KT, kernel thickness; KW, kernel width; PL, plumule length; PRL, primary root length; SL, shoot length; *, $p < 0.05$; **, $p < 0.01$; ***, $p < 0.001$.

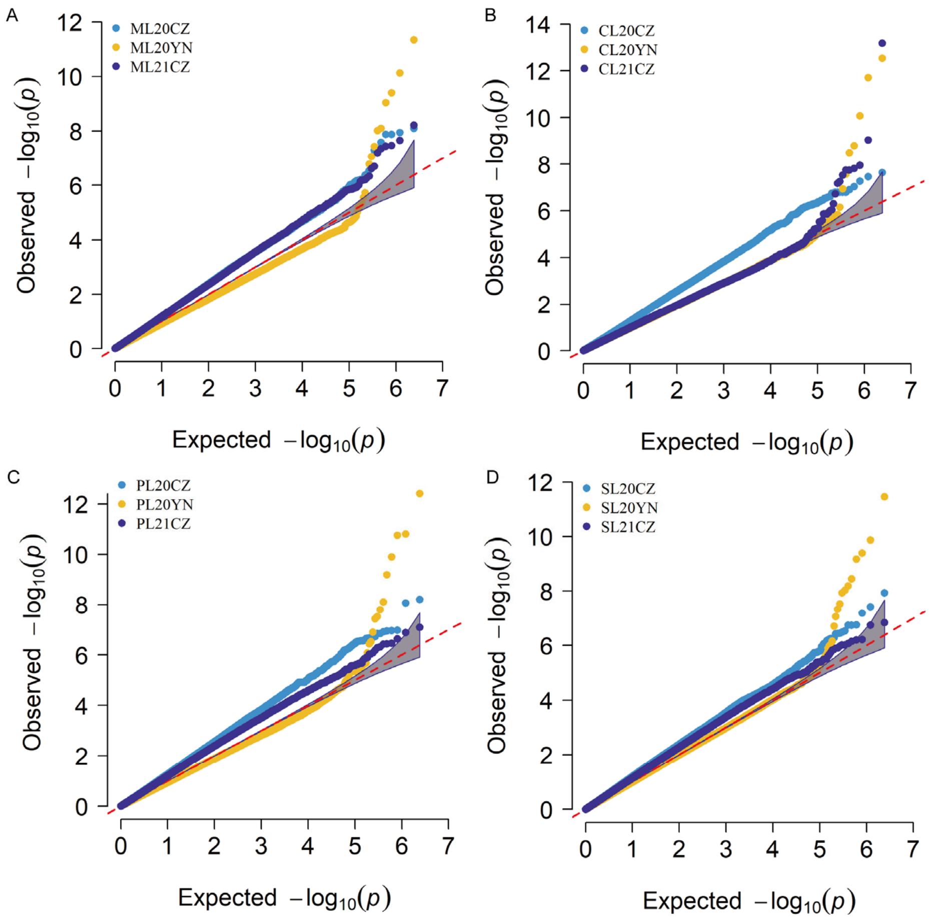

Figure S2. Q-Q plots of the GWAS for deep-sowing tolerance-related traits. (A-D) Q-Q plots of ML, CL, PL, and SL, respectively.

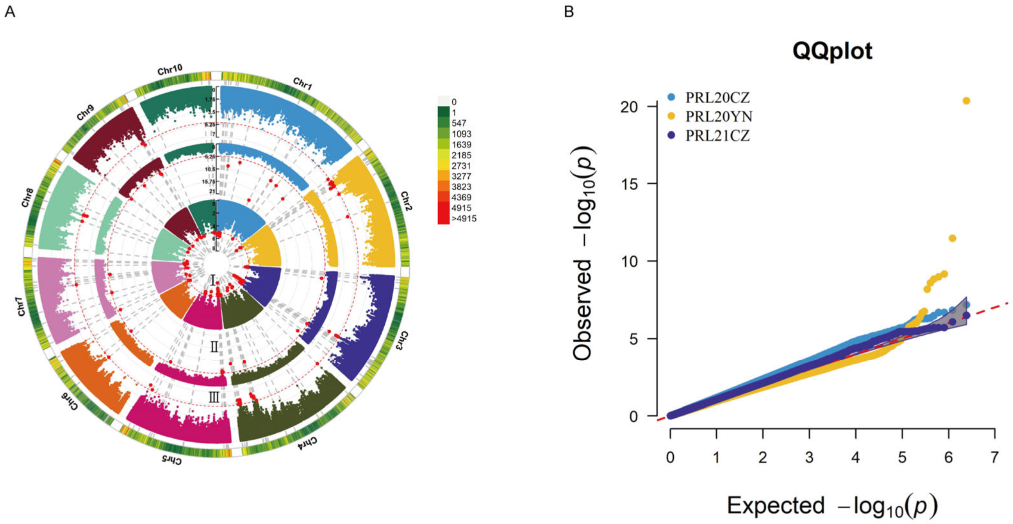

Figure S3. Circular Manhattan and Q-Q plots of the GWAS for PRL. (A) Manhattan plot and (B) Q–Q plots of PRL. I–III represent different environments: (I) 2020 Chongzhou, Sichuan; (II) 2020 Xishuangbanna, Yunnan; (III) 2021 Chongzhou, Sichuan.

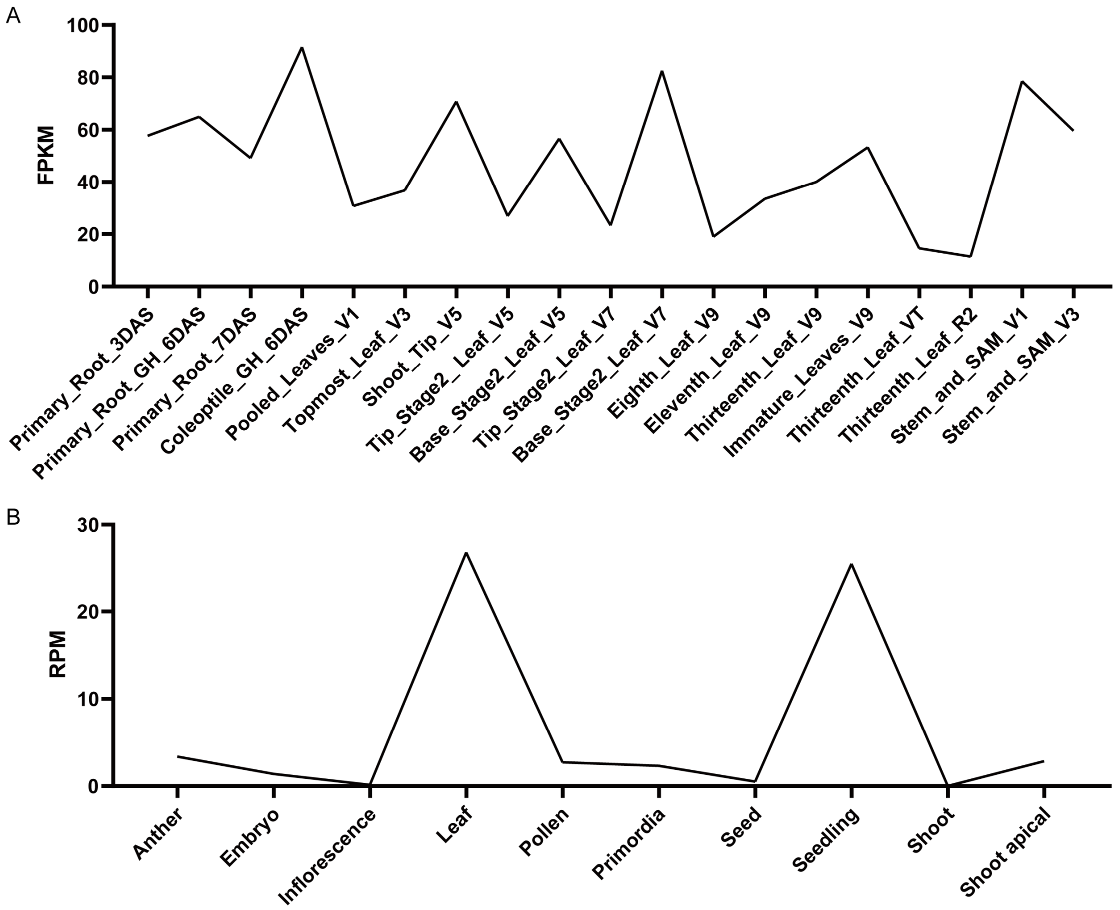

Figure S4. Expression pattern analysis of the candidate genes. (A) Expression pattern analysis of *Zm00001d002644*. DAS, days after sowing; Vn, vegetative stage corresponding to the number of emerged leaves; VT, vegetative tasseling (last branch of tassel fully emerged); R2, reproductive 2; SAM, shoot apical meristem; GPNS, germination paper in nutrient solution; GH, greenhouse. (B) Expression pattern analysis of *zma-MIR171h*.

TABLES:

Table S1. Variance analysis for deep-sowing tolerance-related traits in association populations under three environments.

Trait	*V*g	*V*e	*V*ge	*V*gy	H^2
ML	9.93	4.55	0.58	1.30	0.89
CL	0.82	0.66	0.12	0.10	0.84
SL	21.53	10.41	1.23	3.29	0.88
PL	9.73	5.68	0.60	0.68	0.91
PRL	24.57	40.56	4.21	1.74	0.82

ML, mesocotyl length; CL, coleoptile length; PL, plumule length; SL, shoot length; PRL, primary root length; *Vg*, variance of genotype; *Ve*, variance of residual; *Vge*, the interaction of genotype and environments variance; *Vgy*, the interaction of genotype and year variance; H^2, heritability of trait

Tabel S2. QTLs identified in association population by GWAS.

QTL name	Chr.	Marker	Pos_Start	Pos_End	p-Value	PVE (%)
qML1-1	1	S1_110004943	109994943	110017159	1.62E-06	10.39
		S1_110007159			5.91E-06	9.24
qML1-2	1	S1_194116689	194106689	194126689	6.85E-07	11.76
		S1_194157257			2.01E-06	9.71
qML1-3	1	S1_194158296	194147257	194169247	4.45E-06	8.76
		S1_194159247			5.07E-06	8.31
qML1-4	1	S1_194270755	194260755	194280755	3.25E-06	8.87
qML1-5	1	S1_195524246	195514246	195534246	2.90E-06	9.71
qML2-1	2	S2_10671389	10661389	10681389	2.20E-06	9.06
		S2_12026554			8.94E-07	11.00
qML2-2	2	S2_12026643	12016551	12036658	4.24E-06	9.83
		S2_12026658			1.71E-06	10.44
		S2_18026291			3.55E-06	9.10
qML2-3	2	S2_18026620	18016291	18044787	5.40E-06	8.48
		S2_18026630			5.40E-06	8.48
		S2_18034787			7.70E-06	8.04
qML2-4	2	S2_23203384	23193384	23213384	6.54E-06	5.23
qML2-5	2	S2_43709112	43699112	43726095	5.66E-06	8.49
		S2_43716095			6.83E-07	11.51
qML2-6	2	S2_213606635	213596635	213616635	3.31E-06	8.85
qML3-1	3	S3_12762736	12752736	12772736	6.24E-06	7.45
qML4-1	4	S4_160985432	160975432	160995432	5.37E-06	9.89
qML4-2	4	S4_195793824	195783824	195803824	3.39E-06	8.73
qML6-1	6	S6_142481492	142471492	142491492	6.69E-06	8.20
qML6-2	6	S6_162806776	162796776	162816776	4.45E-06	8.48
qML7-1	7	S7_122227718	122217718	122237718	4.23E-09	12.35
qML8-1	8	S8_109456989	109446989	109466989	2.21E-06	13.71
		S9_3341586			2.63E-06	8.85
		S9_3341926			1.99E-06	9.54
		S9_3341944			4.86E-06	8.72
qML9-1	9	S9_3342271	3331586	3353110	4.09E-06	8.88
		S9_3342277			3.86E-07	10.76
		S9_3342750			4.88E-06	8.93
		S9_3343110			4.09E-06	8.88
qML9-2	9	S9_126616020	126606020	126626020	4.94E-06	8.67
qML10-1	10	S10_72831144	72821144	72841144	3.04E-06	10.19
qML10-2	10	S10_106037965	106027965	106047965	5.21E-06	8.37
qML10-3	10	S10_137270589	137260589	137280589	5.15E-06	9.68
qCL1-1	1	S1_41795664	41785664	41805664	3.03E-06	7.26
qCL1-2	1	S1_258709191	258699191	258719191	2.64E-06	9.65
qCL2-1	2	S2_48090044	4080044	48100044	5.08E-07	9.33
qCL5-1	5	S5_167776704	167766704	167786704	1.27E-06	13.66
qCL8-1	8	S8_172046847	172036847	172056847	1.37E-06	10.28
qCL10-1	10	S10_85280852	85270852	85290852	4.36E-06	19.37
qPL2-1	2	S2_3044013	3034013	3054013	5.63E-08	10.75
qPL2-2	2	S2_18026077	18016077	18036077	2.51E-06	11.81
qPL5-1	5	S5_59821851	59811851	59831851	4.27E-06	11.08
qPL9-1	9	S9_45040998	45030998	45050998	1.36E-06	11.82

qSL2-1	2	S2_18026620<br>S2_18026630	18016620	18036630	9.39E-06<br>9.39E-06	8.64<br>8.64
qSL4-1	4	S4_216138949	216128949	216148949	5.41E-06	9.34
qSL6-1	6	S6_119553252	119543252	119563252	4.66E-06	9.20
qSL8-1	8	S8_105520043	105510043	105530043	3.21E-06	13.77

ML, mesocotyl length; CL, coleoptile length; PL, plumule length; SL, shoot length; PVE, phenotypic variance explained. Pos_Start/Pos_End represents the start and end physical position of the QTL on the chromosome. The QTL names are indicated according to the following rules: the chromosome number is indicated by the first number following the phenotype name, and the number following the dash is used to identify different QTLs found on the same chromosome for the same trait. The marker name indicates chromosome number followed by its physical position.

Tabel S3. List of candidate genes.

Trait	Gene	Annotation
ML	*Zm00001d030167*	Putative ankyrin repeat protein RF_0381
	Zm00001d030166	Glycosyltransferase-like KOBITO 1
	Zm00001d030171	G-type lectin S-receptor-like serine/threonine-protein kinase
	Zm00001d031556	Auxin-induced in root cultures protein 12
	Zm00001d031557	-
	Zm00001d031558	ATP-citrate synthase beta chain protein 1
	Zm00001d002403	Putative peptidyl-tRNA hydrolase PTRHD1
	zma-MIR171h	-
	Zm00001d002644	Gamma-tubulin complex component 5
	Zm00001d003414	NAC domain-containing protein 4
	Zm00001d003415	Cyclic pyranopterin monophosphate synthase, mitochondrial
	Zm00001d047323	Probable magnesium transporter NIPA8
	Zm00001d047324	-
	Zm00001d026057	Zinc finger CCCH domain-containing protein 22
	Zm00001d030168	-
	Zm00001d031553	-
	Zm00001d031555	Cytochrome b561 and DOMON domain-containing protein
	Zm00001d031554	-
	Zm00001d031560	Large ribosomal subunit protein eL32z
	Zm00001d031584	DEAD-box ATP-dependent RNA helicase 5
	Zm00001d031586	-
	Zm00001d031587	Ubiquitin carboxyl-terminal hydrolase 2
	Zm00001d002348	Plasmodesmate-callose-binding protein 3 (PDCB3)
	Zm00001d002349	Ent-kaur-16-ene synthase, chloroplastic
	Zm00001d006656	DNA repair protein RAD51 homolog A
	Zm00001d051510	-
	Zm00001d051511	Protein RGF1-INDUCIBLE TRANSCRIPTION FACTOR 1
	Zm00001d052647	-

	Zm00001d052648	26S proteasome non-ATPase regulatory subunit 14 homolog
	Zm00001d037931	Biotin synthase, mitochondrial
	Zm00001d038699	O-methyltransferase ZRP4
	Zm00001d038698	Auxin response factor 14
	Zm00001d018749	-
	Zm00001d018750	-
	Zm00001d025136	Protein SMAX1-like 4
	Zm00001d020556	WAT1-related protein
	Zm00001d020557	Very-long-chain aldehyde decarbonylase GL1-1
	Zm00001d020558	-
	Zm00001d002809	Mitogen-activated protein kinase 12
	Zm00001d002806	B-box zinc finger protein 21
	Zm00001d039710	Thioredoxin H4-1
	Zm00001d039711	Protein NETWORKED 4B
	Zm00001d010321	Pyruvate, phosphate dikinase 2
	Zm00001d028650	-
	Zm00001d028651	Cysteine-rich repeat secretory protein 55
	Zm00001d028649	-
	Zm00001d033314	Putative B3 domain-containing protein
	Zm00001d016566	Gamma carbonic anhydrase-like 2, mitochondrial
	Zm00001d016567	-
CL	*Zm00001d022600*	Aspartyl protease family protein 1
	Zm00001d024725	Transcription factor MYB2
	Zm00001d023133	-
	Zm00001d023134	-
	Zm00001d012294	Transcription factor LG2
	Zm00001d012295	-
	Zm00001d012296	bZIP transcription factor ABI5 homolog
	zma-MIR171h	-
	Zm00001d002644	Gamma-tubulin complex component 5
	Zm00001d014697	Probable serine/threonine-protein kinase PBL17
	Zm00001d014698	Squamosa promoter-binding-like protein 12
	Zm00001d001659	-
	Zm00001d016136	Putative RING-H2 finger protein ATL12
PL	*Zm00001d017855*	-
	Zm00001d036240	Probable beta-1,3-galactosyltransferase 14
	Zm00001d036241	GDSL esterase/lipase
	Zm00001d008535	-
	Zm00001d008537	-
	Zm00001d045865	Vacuolar protein sorting-associated protein 53 A
	Zm00001d045873	Mannan endo-1,4-beta-mannosidase 6
	Zm00001d036044	Cell cycle checkpoint protein RAD1

	Zm00001d001926	-
	Zm00001d040737	Gibberellin 2-beta-dioxygenase 2
	zma-MIR171h	-
	Zm00001d002644	Gamma-tubulin complex component 5
	Zm00001d053145	Purple acid phosphatase 22
	Zm00001d037290	tRNA threonylcarbamoyladenosine dehydratase 2
SL	*Zm00001d011505*	-
	zma-MIR319c	-
	Zm00001d011504	V-type proton ATPase subunit E
	Zm00001d010236	Cytochrome c
	Zm00001d010237	-
	Zm00001d010238	-

ML, mesocotyl length; CL, coleoptile length; PL, plumule length; SL, shoot length.

Table S4. Pedigree and source information of inbred lines in association population.

Lines	Pedigree	Origin	Sub-populations
BT1	8085×Thai Hybrid	China	Mixed
Dan599	American Hybrid P78599	China	PA/PB
Nan21-3	Jugoslavian Hybrid	China	SS
SW1611	Suwan2	China	Mixed
Shen137	American Hybrid 6JK611	China	PA/PB
647	Landrace	China	Mixed
ZZ13	Unknown	China	SPT
DH3732	DH3732-1-1-2-2-1-1	China	Mixed
CY72	ImProved from Landrace	China	NSS
Sy3073	Syn.D.O	China	NSS
Dan4254	Unknown	USA	Mixed
XZ698	XinZhong698-3-1-1-2-1-2-3	China	PA/PB
Gy237	AIHO	China	NSS
Gy462	AIHO	China	NSS
L3180	Foreign hybrid	China	Mixed
TY11	TY30331-2-8-1-2-1-1	China	Mixed
GEMS9	2111-01_DK212T_S11_F2S4_9166-Blk31/00	USA	SS
LY042	Unknown	China	Mixed
CF3	Unknown	China	SS
Zong31	Synthetic	China	NSS
Ry737	RYD	China	NSS
GEMS21	2152-02_DK888_S11_F2S4_65/97-Blk/97-99	USA	SS
DSB	Landrace	China	SPT
177	Unknown	China	PA/PB
FCD0602	Unknown	China	SS
B113	BS11(FR)C9	China	Mixed
835b	U8112×Ye515	China	PA/PB
D047	Landrace	China	SPT
Ye515	(HuaFeng100×AiGo352)×HuangZaoSi	China	Mixed
MO113	Unknown	China	NSS
By813	BHO	China	NSS
B151	Unknown	China	SPT
Yan414	HuangZaoSi	China	Mixed

By855	BHO	China	NSS
GEMS54	ARI16035:S02-450-1-B	USA	Mixed
Liao159	Unknown	China	Mixed
5237	Dan340×HuangZaoSi	China	SPT
GEMS46	CH05015:N15-8-1-B-B	USA	Mixed
GEMS52	CUBA164:S2008a-326-1-B	USA	SS
9782	American Hybrid	China	PA/PB
GEMS51	CUBA164:S2008a-83-1-B	USA	SS
WH413	HuangZaoSi×WenQing1331	China	SPT
Zheng30	Zheng20×Ye478	China	PA/PB
Ye52106	(Ye1075×Ye106)×AiJin525	China	NSS
WMR	Landrace	China	SPT
Cheng698	Foreign Hybrid	China	Mixed
Q1261	ImProved from K12	China	SPT
GEMS64	CUBA164:S1511b-249-1-B-B	USA	SS
HSBN	Landrace	China	SPT
GEMS6	2088-01_DK212T_S11_F2S4_9157-Blk29/00	USA	SS
A619	The University of Illinois, USA	USA	NSS
GEMS65	Unknown	USA	SS
04K5686	Landrace	China	SPT
Liao5114	Tie7922×Shen5003	China	SS
7381	Unknown	China	PA/PB
GEMS62	AR16026:S17-16-1-B-B	USA	SS
GEMS4	2084-02_DK212T_S11_F2S4_9151-Blk38/00	USA	SS
Si273	American Hybrid	China	Mixed
Sy999	Syn.D.O	China	NSS
Tie7922	American Hybrid 3382	China	SS
Lx9801	Xi502×H21	China	SPT
By809	BHO	China	NSS
By4944	BHO	China	NSS
GEMS19	2146-01_DK888_S11_F2S4_9196-Blk29/00	USA	SS
MO17	CI 187-2/C103	China	NSS
Sy1077	Syn.D.O	China	Mixed
Ry697	RYD	China	NSS
1323	Unknown	China	Mixed
GEMS47	UR13085:N0125-014-001	USA	NSS
JH96C	Synthetic	China	Mixed
GY386B	Unknown	USA	NSS
ZZ03	Unknown	China	PA/PB
HB	Huojiabaimaya	China	Mixed
GEMS48	DKXL370:N11a20-31-1-B-B	USA	NSS
GEMS59	SCROI:N1310-398-1-B	USA	NSS
GEMS50	DKB844:S1601-517-1-B	USA	SPT
Dan3130	American Hybrid P78599	China	NSS
Zheng28	Ye478×Tuxpeno	China	Mixed
Liao138	ImProved from Dan340	China	PA/PB
501	Unknown	China	PA/PB
B77	B77-3	China	Mixed
Wu109	XiDan7	China	Mixed
Gy220	AIHO*	China	NSS
GEMS5	2086-01_DK212T_S11_F2S4_9154-Blk20/00	USA	SS
By807	BHO	China	NSS
Zheng32	American Single-cross 3382	China	SS
GEMS63	CUBA164:S1511b-325-001	USA	SS

GEMS44	CH05015:N12-140-1-B-B	USA	Mixed
Si446	Qu43×Zi330	China	NSS
TY8	TY30331-2-7-1-2-1-1	China	Mixed
GEMS23	2156-02_DK888_S11_F2S4_h92847-Blk13/00	USA	SS
LK11	Mo17×Zi330	China	SPT
9642	Unknown	China	NSS
Liao5263	Liao526-2-2-2-1-3-1	China	Mixed
5213	ImProved from Mo17	China	SPT
GEMS37	PE001n16F2S2-181	USA	NSS
NMJT	Landrace	China	Mixed
Yu374	Synthetic	China	NSS
GEMS16	2132-03_DK888_S11_F2S4_9187-Blk22/00	USA	SPT
NX15	Landrace	China	Mixed
Chang7-2	HuangZaoSi×WeiChun	China	SPT
X1141P	X1141P-1-4-6-1-3-2-1-1	China	NSS
Shen135	Unknown	China	PA/PB
GEMS15	2131-01_DK888_S11_F2S4_9184-Blk20/00	USA	SS
4wv	Unknown	USA	SS
JA96C	Unknown	USA	Mixed
A188	4-29(Silver King) x 46(N.W. Dent)4^4 N.W	USA	SPT
FAPW	B14AH×B37H .	USA	SS
LH132	(H93×B73)×B73	USA	SS
HBA1	Pioneer 3195 hybrid×Pioneer 3199 hybrid	USA	SS
2369	(2702H×B73)×B73	USA	SS
PhG71	A632Ht×207 specifically A632/207)×42112143	USA	Mixed
PhG83	814×207 specifically 814/207)×44××××	USA	IDT
LH82	610×LH7	USA	Mixed
LH150	Pioneer 3147 hybrid	USA	SS
B47	B37×SD105 specifically B37<3-××#-SD105-#)F21323×11×	USA	SS
Q381	One off-type plant in a planting of Pioneer 3369 hybrid test plot planting was selected an selfed	USA	IDT
FBHJ	#(FBAB×B84)×B84	USA	SS
PhH93	PH806×207 specifically PH806/207)×62111	USA	IDT
PhT10	B73×G39 specifically B73/G39)×61113×	USA	SS
PhW79	PHT90×PH595 specifically PHT90/PH595)×211××	USA	PA/PB
PHW03	801×G48 specifically 801/G48)41221×	USA	NSS
P508F	Unknown	USA	Mixed
PHW43	995×G35 specifically 995/G35)×33311×	USA	SS
PHW57	Unknown	USA	IDT
IBC2	J6×(J6×Mo17Ht) specifically J6"Mo17Ht-7221	USA	IDT
IBB15	J6×(J6×W70884) specifically J6"W70884-4223B	USA	SS
L135	Pioneer 3901 hybrid×W117	France	NSS
LP1NRHT	Unknown	USA	SS
DJ7	(B73×BS16) backcrossed to B73 three times	USA	SS
L9-1391	Unknown	USA	NSS
PB80	(1067-1×B73)×(B73Ht.1 BC6)	USA	SS
PhT77	814×995 specifically 814/995)×8111××	USA	Mixed
NV790	Unknown	USA	SS
P508M	Unknown	USA	SS
P128M	Unknown	China	SS

69-55	Unknown	China	SS
LH198	LH132×(LH132×B84)	USA	SS
5553	Unknown	China	Mixed
NV790	Unknown	USA	SS
Zheng29	Shen5003	China	NSS
Qi319	American Hybrid P78599	China	PA/PB
Gy386	AIHO	China	NSS
150	Unknown	China	Mixed
BZN	Landrace	China	NSS
TY3	TY30331-2-4-2-2-2-1	China	Mixed
Yu87-1	American Hybrid	China	PA/PB
Ye8001	Ye488×3189	China	PA/PB
IRF314	(PA91/LH98A)-6-420-64-2-1-3-1-1-1	China	NSS
GEMS58	ARI01150:N04-696-1-B	USA	NSS
Shen5003	American Single-cross 3147	China	PA/PB
GEMS31	2282-01_XL380_S11_F2S4_9226-Blk26/00	USA	SS
384-2	Unknown	China	Mixed
Dan340	Lv9×Wide Pod Corn	China	NSS
GEMS30	2258-03_XL380_S11_F2S4_71/97-Blk/98	USA	Mixed
8902	Ye107×81162	China	Mixed
Z2018F	Zheng2018F2*698-3-1-2-1-1	China	Mixed
J4112	A619×U8112	China	Mixed
Ji53	Derived from Ji Synthetic C0-2	China	NSS
GEMS11	2116-02_DK212T_S11_F2S4_9172-Blk28/00	USA	SS
GEMS2	Selected from GEM FS8(A)S:S09 population	USA	SS
EN25	Unknown	China	Mixed
238	Unknown	China	NSS
GEMS14	2127-01_DK888_S11_F2S4_9181-Blk21/00	USA	SS
JH59	American Hybrid P78599	China	NSS
LXN	Landrace	China	Mixed
Dan360	Lv9×Wide Pod Corn	China	Mixed
By4839	BHO	China	NSS
Xun971	Landrace	China	Mixed
Zheng22	Dan340×E28	China	SS
Sy1035	Syn.D.O	China	NSS
R08	American Hybrid P78641	China	Mixed
Zheng653	(Shen5003×Zong31)×Shen5003	China	Mixed
Zhi41	Unknown	China	NSS
Liao5262	Liao526-2-2-2-1-2-1	China	Mixed
303WX	Landrace	China	SPT
05W002	05W002-1	China	Mixed
HZS	TangSiPingTou	China	SPT
TY10	TY30331-2-8-1-1-3-2	China	Mixed
TY5	TY30331-2-4-2-2-3-1	China	SS
GEMS29	2253-01_XL370A_S11_F2S4_9220-Blk24/00	USA	SS
Zheng35	Unknown	China	Mixed
GEMS25	2226-02_XL370A_S11_F2S4_9211-Blk25/00	USA	Mixed
Dan9046	Shen5003×Tie7922	China	NSS
ES40	Landrace	China	SPT
975-12	78698×Dan9046	China	PA/PB
ZaC546	Variant plants from C103	China	NSS
GEMS1	(Piura 144:PI 503806 x B94)\\\B94 S1	USA	Mixed
LG001	Unknown	China	PA/PB

04K5672	Landrace	China	Mixed
GEMS32	2283-01_XL380_S11_F2S4_9229-Blk20/00	USA	PA/PB
ZB648	ZhongBei648-1	China	Mixed
Ye488	U8112×Shen5003	China	PA/PB
GEMS12	2120-01_DK888_S11_F2S4_9175-Blk28/00	USA	PA/PB
GEMS49	DKB844:S1601-512-1-B	USA	SS
GEMS35	PE001n16F2S2-172	USA	NSS
Ry713	RYD	China	Mixed
07KS4	Unknown	China	Mixed
7884-4Ht	78-6×H84	China	Mixed
M165	Unknown	China	Mixed
Lv28	LvDaHongGu	China	NSS
B111	BSSS(R)C9	China	Mixed
BS16	Population BS16	China	SPT
M97	Unknown	China	Mixed
4F1	ImProved from Mo17 by radiation	China	NSS
GEMS36	PE001n16F2S2-176	USA	Mixed
Ji846	Ji63×Mo17	China	NSS
TX5	Landrace	China	Mixed
M153	Unknown	China	Mixed
GEMS27	2250-01_XL370A_S11_F2S4_9214-Blk21/00	USA	Mixed
7327	S37×3H-2	China	NSS
GEMS13	2121-04_DK888_S11_F2S4_9178-Blk29/00	USA	SS
IRF291	A671×LH38	China	NSS
DH29	American Hybrid P78599	China	SPT
P178	American Hybrid P78599	China	PA/PB
04K5702	Landrace	China	SPT
Si434	Si466×Hua94	China	SS
H99	Illinois Synthetic 60C	USA	Mixed
6wv	Unknown	USA	SS
1371	Unknown	USA	SS
1373	Unknown	USA	SS
4676A	1067-1×(B-line Composite)	USA	SS
LH149	((A662×B73 S1)×B73)×B73	USA	SS
PhW65	861×595 specifically 861/595)×8211131×	USA	Mixed
W8304	(B14A×B73)×B73	USA	Mixed
NS501	A634×K1	USA	IDT
NV792	Unknown	USA	SS
LH82R	Unknown	USA	NSS
LH52	(Mo17×(610×Mo17))×Mo17	USA	NSS
NV794	Unknown	USA	SS
NV740	Unknown	USA	NSS
207	Unknown	USA	IDT
PhG72	891×207 specifically 891/207)×4123×	USA	IDT
NQ508	Pioneer 3713 hybrid	USA	IDT
LH143	(A635Ht×A632Ht)×A632Ht.	USA	SS
PHR25	B83×207 specifically B83/207)×242211111×	USA	IDT
PHK29	B47×AC54 specifically B47/AC54)N×711233×	USA	IDT
PHN29	G69×G40 specifically G69/G40)×A6211	USA	IDT
CR14	A synthetic composed of high yielding selections derived from the selfed hybrid (B73Ht×CM105) and the selfed hybrid (B73Ht×CQ187)	USA	SS
GM502	Unknown	USA	NSS
J8606	P101×C103G	USA	IDT

W8555	B73Ht/B84	USA	SS
2139	Unknown	China	Mixed
F42	Mutation induction of B73 with nitrosoquanidine.	USA	SS
LH57	(Mo17×H99)×LH53	USA	NSS
LH60	LH55×LH47	USA	NSS
LP5	(Glamos×B73Ht)×B73Ht	France	SS
PhK42	207×(207×806) specifically 207<2806)C031344×	USA	IDT
H8431	(377×B386)×347	USA	SS
LH39	Oh43×L120	USA	NSS
78004	B73×A634	USA	SS
LH185	LH59×LH123Ht	USA	NSS
LY88M	Unknown	USA	SS
BN501	Unknown	China	PA/PB
LH146HT	(B73×CM105)×CM105	USA	NSS
